# Pneumolysin Is Responsible for Differential Gene Expression and Modifications in the Epigenetic Landscape of Primary Monocyte Derived Macrophages

**DOI:** 10.3389/fimmu.2021.573266

**Published:** 2021-05-11

**Authors:** Joby Cole, Adrienn Angyal, Richard D. Emes, Tim John Mitchell, Mark J. Dickman, David H. Dockrell

**Affiliations:** ^1^ Department of Infection, Immunity and Cardiovascular Diseases, University of Sheffield, Sheffield, United Kingdom; ^2^ Sheffield Teaching Hospitals NHS FT, Sheffield, United Kingdom; ^3^ The Florey Institute, University of Sheffield, Sheffield, United Kingdom; ^4^ Department of Chemical and Biological Engineering, University of Sheffield, Sheffield, United Kingdom; ^5^ Advanced Data Analysis Centre, University of Nottingham, Nottingham, United Kingdom; ^6^ School of Veterinary Medicine and Science University of Nottingham, Nottingham, United Kingdom; ^7^ Institute of Microbiology and Infection, University of Birmingham, Edinburgh, United Kingdom; ^8^ MRC Centre for Inflammation Research, University of Edinburgh, Edinburgh, United Kingdom

**Keywords:** histone post translational modifications, *Streptococcus pneumoniae*, pneumolysin, tumour necrosis factor, monocyte derived macrophages

## Abstract

Epigenetic modifications regulate gene expression in the host response to a diverse range of pathogens. The extent and consequences of epigenetic modification during macrophage responses to *Streptococcus pneumoniae*, and the role of pneumolysin, a key *Streptococcus pneumoniae* virulence factor, in influencing these responses, are currently unknown. To investigate this, we infected human monocyte derived macrophages (MDMs) with *Streptococcus pneumoniae* and addressed whether pneumolysin altered the epigenetic landscape and the associated acute macrophage transcriptional response using a combined transcriptomic and proteomic approach. Transcriptomic analysis identified 503 genes that were differentially expressed in a pneumolysin-dependent manner in these samples. Pathway analysis highlighted the involvement of transcriptional responses to core innate responses to pneumococci including modules associated with metabolic pathways activated in response to infection, oxidative stress responses and NFκB, NOD-like receptor and TNF signalling pathways. Quantitative proteomic analysis confirmed pneumolysin-regulated protein expression, early after bacterial challenge, in representative transcriptional modules associated with innate immune responses. In parallel, quantitative mass spectrometry identified global changes in the relative abundance of histone post translational modifications (PTMs) upon pneumococcal challenge. We identified an increase in the relative abundance of H3K4me1, H4K16ac and a decrease in H3K9me2 and H3K79me2 in a PLY-dependent fashion. We confirmed that pneumolysin blunted early transcriptional responses involving TNF-α and IL-6 expression. Vorinostat, a histone deacetylase inhibitor, similarly downregulated TNF-α production, reprising the pattern observed with pneumolysin. In conclusion, widespread changes in the macrophage transcriptional response are regulated by pneumolysin and are associated with global changes in histone PTMs. Modulating histone PTMs can reverse pneumolysin-associated transcriptional changes influencing innate immune responses, suggesting that epigenetic modification by pneumolysin plays a role in dampening the innate responses to pneumococci.

## Introduction

Pneumolysin (ply) is one of the key virulence factors of *S. pneumoniae* (the pneumococcus), the leading cause of community-acquired pneumonia (1) and is present in the majority of clinical isolates causing invasive pneumococcal disease (IPD) (2, 3). Pneumolysin is a cholesterol-dependent cytolysin and part of a family of toxins expressed in Gram-positive bacteria (4). It is a 53 kDa protein that contains four domains (5). One mechanism of action is through pore formation (6) but increasingly it is recognized to mediate additional actions independent of the ability to form pores (7).

In murine models of bacteraemia ply sufficient mutants are associated with increased lethality compared to ply deficient mutants, linking the toxin to virulence (8). Furthermore, the transmission of *S. pneumoniae* between hosts has been linked to the presence of inflammation in the nasopharynx and pneumolysin has been shown to promote inflammation, increase transmission and foster the survival *ex vivo* of *S. pneumoniae* (9). It has been also suggested that pneumolysin facilitates blood stream invasion by *S. pneumoniae* (3). This highlights its importance as a key virulence factor of *S. pneumoniae* due to its role in the transmission of *S. pneumoniae* between hosts, in the progression from nasopharyngeal colonisation to invasive pneumococcal disease, the stimulation of inflammation and its cytotoxic effects (1).

Pneumolysin has been shown to alter a variety of immune responses. For example it has been demonstrated to both activate the classical complement pathway (10) as well as play a role in complement evasion (11). Pneumolysin has been associated with stimulation of NRLP3 and potentially other inflammasome components (12, 13). In human monocytes it is associated with production of tumour necrosis factor alpha (TNF-α) and interleukin (IL-)1β (14). Pneumolysin has been shown to be responsible for the differential expression of multiple genes in undifferentiated THP-1 cells, a monocytic cell line (15), but its impact on gene expression in primary macrophages has not been established in detail. More recently, pneumolysin has been shown to bind the mannose receptor C type 1 in mouse alveolar macrophages leading to diminished pro-inflammatory cytokine release and enhanced bacterial survival (16).

The subversion of the host immune system is one of the key components of bacterial pathogenesis. Microorganisms can highjack host gene expression to their benefit (17–19). Epigenetic mechanisms such as histone post-translational modifications (PTMs) have been shown to regulate gene transcription (20, 21). Moreover, they can be modulated by a number of different bacterial components (22). Experiments using both *Legionella pneumophila* (19), and *Listeria monocytogenes* (17) have shown that bacterial interaction with the THP-1 monocytic cell line also modifies histone PTMs. Furthermore, *H. pylori* has been shown to alter both the host epigenome and transcriptome (23). In the case of *Listeria monocytogenes*, Listeriolysin O, a pore-forming cytolysin similar to ply, is secreted causing dephosphorylation at serine 10 of histone H3 and reduction in the levels of acetylated H4 in THP-1 cells. In HeLa cells these changes altered the transcriptional profile, which was associated with a decrease in IL-6 and other genes involved in innate immune responses (18). More recently, *Streptococcus pneumoniae via* the action of ply was shown to dephosphorylate serine 10 on Histone H3 mediated by host cell phosphatase PP1 (24). which in turn led to efficient infection of epithelial cells. Furthermore, *S. pneumoniae* has been shown to lead to chromatin remodelling in an epithelial cell model in a KDM6B dependent manner (25). This supports the hypothesis that bacteria alter the epigenetic profile of the host cell as a strategy for immune subversion, limiting the inflammatory response to increase their survival.

The consequences of epigenetic changes during acute bacterial infections are, however, not fully understood. The aim of this study was to investigate the consequences and mechanism underpinning the ability of pneumolysin to modulate translational responses to *S. pneumoniae* using primary human macrophages. We found that pneumolysin modulates a broad range of immune transcriptional responses in macrophages and differential protein expression analysis also confirmed changes in key transcriptional modules. Moreover, we identified global changes in the abundance of histone PTMs in MDMs in a pneumolysin dependent manner. We illustrate that one key pneumolysin-regulated immune response, early TNF-α production is also altered by chemical manipulation of histone PTMs.

## Materials and Methods

### Bacterial Strains

*Streptococcus pneumoniae* serotype 2 strain D39, the isogenic pneumolysin-deficient mutant D39-Δ*ply* (Δ*ply*), which has a single amino acid substitution in the pneumolysin sequence generating a STOP codon and the reconstituted mutant *ply*-D39-Δ*ply*, which expresses ply under an erythromycin promoter, were kindly obtained from Prof T. Mitchell (University of Birmingham). These were cultured and characterized as previously described (7). Strains were grown in brain heart infusion broth and 20% FCS to mid exponential phase (with or without 1μg/mL erythromycin).

### MDM Infection

Whole blood was obtained from healthy volunteers. Ethical approval was granted by South Sheffield Regional Ethics committee (07/Q2305/7). Peripheral blood mononuclear cells were separated by differential centrifugation using a Ficoll-Paque gradient and differentiated into monocyte derived macrophages (MDM) for 14 d as previously described in 24 well plates (Corning) (26). Bacteria were washed in PBS and re-suspended in RPMI 1640 supplemented with 10% pooled human immune serum (from previously vaccinated volunteers with demonstrable antibody levels to serotype 2 pneumococci) (27). MDM were challenged with either opsonised *S. pneumoniae*, Δ*ply* or PBS, at a MOI of 10, rested on ice for 1 h and incubated at 37°C in 5% CO_2_ for a further 3 h (28). For certain experiments cells were treated with 3 μM vorinostat (SAHA, Sigma) or 0.5% DMSO (vehicle control) for 30 min prior to bacterial challenge and vorinostat reintroduced after bacterial challenge.

### *S. pneumoniae* Internalisation Assay

MDM were challenged with opsonized *S. pneumoniae* for 3* h* then washed three times in PBS, incubated for 30 min in RPMI media (Lonza) with 40 units/mL of benzylpenicillin (Sigma) and 20 mg/mL gentamicin (Sanofi). The cells were then washed three times in PBS and incubated in 250 μL of 2% saponin (Sigma) for 12 min at 37°C in 5% CO_2_, then 750 μL of PBS was added, followed by vigorous pipetting. The number of internalised viable bacteria were measured by counting the number of colony forming units on Colombia blood agar (CBA) after 24 h incubation at 37°C in 5% CO_2_ contained in these lysates measured in triplicate.

### Cytokine Measurements

Supernatants were obtained from MDM challenged with bacteria and were analysed as per the manufacturers protocol using either Tumour necrosis factor alpha (TNF-α (Ready-set-go!™, eBioscience)) or interleukin 6 (IL-6kit (Ready-set-go!™, eBioscience)). Briefly, 96 well ELISA plates were coated with 100 μL of 1x capture antibody overnight at 4°C, washed, and blocked in 200 μL assay diluent for 1 h at room temperature. After a wash, the supernatants were then added as were the standards (recombinant human TNF-α or recombinant human IL-6) and incubated for 2 h at room temperature. The wells were then washed and the detection antibody (biotin-conjugated anti-human TNF-α or anti-human IL-6) was added. After washing 100 μL avidin-horse radish peroxidase (HRP) was added to each well for 30 min at room temperature. Plates were then washed, prior to adding 100uL tetramethylbenzidine substrate solution for 15 min at room temperature. The reaction was stopped by adding 2M sulphuric acid, the plate was then read at 450 nm using a Multiskan® EX plate reader (Thermo Scientific), and the data analysed in GraphPad Prism version 7.0c. (GraphPad Software).

### RNA Extraction

After 3 h of bacterial challenge, cells were washed and lysed in 600 μL Tri Reagent ^®^ (Sigma) for 15 mins at room temperature before storing at -80 C. Ribonucleic acid (RNA) extraction was performed following the manufacturers guidelines for Direct-Zol™ RNA miniPrep (Zymo). Briefly, samples in Tri Reagent^®^ were centrifuged at 12 000*g* for 1 min, then the supernatant was transferred to a fresh 1.5 mL tube, 100% ethanol was added in a 1:1 ratio and well mixed. This was then transferred to the Zymo-spin™ column, centrifuged for 1 min, then washed using 400 μL Direct-Zol™ RNA pre-wash, centrifuged again at 12 000*g* for 1 min, then washed in 700 μL RNA wash buffer and again centrifuged at 12 000*g* for 1 min. Finally the RNA was eluted out in 50µl DNase/RNase free water.

### Microarray mRNA Expression Analysis

Affymetrix chip micro-array (Human Genome U133 plus 2.0 array, Santa Clara, CA) analysis of samples from three individuals was undertaken to characterise gene expression as previously described (29). Briefly, CEL files were analysed using R version 3.4.0. CEL files were read using *Simpleaffy* version 2.52.0 (30); background intensity correction, median correction and quantile probeset normalisation was performed using robust multi-array average expression with the help of probe sequence (GCRMA), using *AffyPLM* version 1.52.1 (31). The quality control matrix was generated using *Simpleaffy* and *AffyPLM*. Principal component analysis was performed in R. The probes whose intensity was within the lowest 20^th^ centile were removed, using Dplyr version 0.7.1. Differential gene expression was calculated using *Limma* version 3.32.2.

### Pathway Analysis

Pathway analysis was performed from the differentially-expressed gene lists generated. Hypergeometric tests were calculated in R, using *GO.db* version 3.4.1 to search the Gene Ontology (GO) database (32) for molecular function, cellular component, and biological process, using a p value cut off of 0.01 and a minimum of 3 genes. In addition, canonical pathway analysis was performed using XGR (1.1.5) (33) using all of the differentially expressed probes identified by ANOVA calculated in *Limma* as the test background.

### Real Time Quantitative Polymerase Chain Reaction (RT-qPCR)

The abundance of TNFα mRNA in bacterial exposed and mock-infected MDM was measured using qPCR. To perform cDNA synthesis a high-capacity cDNA reverse transcription kit (Applied Biosystems) was used to make complementary DNA for qPCR assay as per manufacturer’s protocol. The DNA products were quantified on QuantStudio5 (Abi) using GoTaq qPCR master mix (Promega). The reactions were prepared as per manufacturer’s protocol.

Fold change was calculated using Delta Delta Ct values.

Primers used:
* TNFα*: Forward 5' CTCTTCTGCCTGCTGCACT
          TG 3'
     Reverse 5' ATGGGCTACAGGCTTGTCAC
          TC 3'
* GAPDH:* Forward 5' TGCACCACCAACTGCTTA
          GC 3'
     Reverse 5' GGCATGGACTGTGGTCATG
          AG 3'
* Actin:* Forward 5' CCTTTGCCGATCCGCCG 3'
     Reverse 5' GATATCATCATCCATGGTGAG
           CTGG 3'
* PTGS2* Forward 5’ CGGTGAAACTCTGGCTAGG
          GA 3'
     Reverse 5' GCAAACCGTAGATGCTCAGG
          GA 3'
* C-JUN*  Forward 5' CCTTGAAAGCTCAGAACTCGG
          AG 3'
     Reverse 5' TGCTGCGTTAGCATGAGTTG
          GC 3'
* IL1RN* Forward 5’ ATGGAGGGAAGATGTGCCTG
          TC 3'
     Reverse 5' GTCCTGCTTTCTGTTCTCGC
          TC 3'
* NR4A2*  Forward 5’ AAACTGCCCAGTGGACAAGC
       GT 3'
     Reverse 5' GCTCTTCGGTTTCGAGGGCA
          AA 3'

### Quantitative Proteomics

Protein lysates were digested with trypsin in conjunction with the Filter aided separation protocol (FASP) (34). Briefly, cells were lysed in 150 µL of 4% Sodium dodecyl sulphate (Sigma), 100 mM Tris HCl (Sigma) pH 7.6, 0.1 M Dithiothreitol (Sigma) and quantified using a BioRad DC assay as per the manufacturer’s protocol. 100 µg of protein lysates were mixed with 200 µL of 8 M urea dissolved in triethylammonium bicarbonate (TEAB) and added to the filter mounted in 11.5 mL low-bind Eppendorf. The tubes were centrifuged at 14 000 g for 30 min, the flow through discarded and further two 200 µL washes in 8M urea were performed. Then 100 µL 0.5 M iodoacetic acid was added for 20 min at room temperature and then centrifuged at 14 000 g for 30 min. The flow-through was discarded. The membrane was washed three times with 100 µL of 8 M urea, followed by three washes 100 mM TEAB. The lysates were trypsin digested overnight at 37°C. The digested proteins were eluted in 120 µL 100mM TEAB. The samples were then desalted using Hypersep Hypercarb™ (ThermoScientific) tips following the manufacturer’s protocol. Briefly, tips were primed with elution solution (60% Acetonitrile 0.1% TFA) then washed in 0.1% TFA. The sample was then re-suspended on the tip by pipetting up and down 50 times. The tip was cleaned in 0.1%TFA, eluted 60% ACN 0.1% TFA and then 90% ACN 0.1% TFA. The samples were then dried down in Speedvac (Eppendorf). The peptides were re-suspended in 0.1%TFA and 3% ACN and loaded into and run on Ultimate 3000 RSLC nano flow liquid chromatography system with a PepMap300 C18 trapping column (Thermo Fisher), coupled to Q-Exactive HF Orbitrap mass spectrometer (Thermo Fisher). Peptides were eluted onto a 50 cm x 75 μm Easy-spray PepMap C18 column with a flow rate of 300 nL/min as previously described (35). Peptides were eluted using a gradient of 3% to 35% solvent B over 75 min. Solvents were composed of 0.1% formic acid (FA) and either 3% acetonitrile (ACN) (Solvent A) or 80% ACN (Solvent B). The loading solvent was 0.1% TFA and 3% ACN. Data acquisition was performed in full scan positive mode, scanning 375 to 1500m/z, with an MS1 resolution of 120 000, and AGC target of 1x10^6^. The top 10 most intense ions from MS1 scan were selected for Collision induced dissociation. MS2 resolution was of 30 000 with AGC target of 1x10^5^ and maximum fill time of 60 ms, with an isolation window of 2 m/z and scan range of 200-2000 m/z and normalised collision energy of 27.

### MaxQuant Data Analysis

The raw data from the MS were analysed in MaxQuant (version 1.5.6.5). The database search engine Andromeda was used to search the spectra against the UniProt database. The search settings were as follows: trypsin/P digestion, with up to 2 missed cleavages, fixed modification was carbamidomethyl (C), variable modifications were oxidation (M) and acetylation (Protein N-term), Label Free Quantification (LFQ) was performed with a minimum number of neighbours of 3 and average number of neighbour of 6. Peptide tolerance was set at 4.5 ppm and minimum peptide length was set at 7, amino acid maximum peptide mass was set at 4600 Da and Protein FDR was set at 0.01. Downstream analysis was performed in R version 3.4.0. The protein identification files were read in, results matching to a reverse sequence database and or those matching to a contaminant database were removed as were those with less than 2 unique peptides. The label-free intensities were then median-corrected for each sample and log2 transformed. Differential protein expression was calculated using Limma version 3.32.2. a repeated measures ANOVA to enable comparison to the microarray analysis (36).

### Histone Extraction

Histone extraction was performed as previously described (37). Briefly, MDMs were washed in PBS and scrapped in ice cold PBS with 1x protease inhibitors (Roche Complete EDTA free), before being pelleted at 900*g* for 10 min. Cell pellets were lysed in hypotonic lysis solution then re-suspended in 400 µL 0.2 M H_2_SO_4._ The histones were precipitated out by adding 132uL of 6.1N TCA to the supernatant and washed in acetone then resuspended in 100 μL of water (HPLC grade). Samples then underwent chemical derivatisation as previously described (38). 10 μL of 100 mM ammonium bicarbonate pH 8 and 4 μl of ammonium hydroxide was added to 10 μg of histone sample. Then 10 μL of propionic anhydride in isopropanol (1:3 ratio) was added and 100% ammonium hydroxide used to keep the pH >8.0. The sample was incubated at 37°C for 15 min. Then it was dried down in a vacuum centrifuge (Concentrator plus, Eppendorf) and the process repeated. The samples were re-suspended in 40 μL of 100 mM ammonium bicarbonate and then tryptically digested overnight. The digestion was stopped by addition of glacial acetic acid and freezing at -80°C for 5 min. Finally, the samples were dried down before undergoing a further two rounds of proprionylation. Hypersep™ Hypercarb™ tip were used to desalt the samples of the chemical derivatisation residues following the manufacturer’s protocol for Hypersep ™ Hypercarb ™ (ThermoScientific) (28).

### Quantitative MS Analysis of Histone PTMs

The histone samples were re‐suspended in 0.1% trifluoroacetic acid (TFA) and were analysed on an Ultimate 3000 online nano‐LC system with a PepMap300 C18 trapping column (ThermoFisher Scientific), coupled to a Q Exactive HF Orbitrap (ThermoFisher Scientific). Peptides were eluted onto a 50 cm × 75 μm Easy‐spray PepMap C18 analytical column at 35°C. Peptides were eluted at a flow rate of 300 nL/min using a gradient of 3% to 25% over 55 min then 25% to 60% until 81 min. Solvents were composed of 0.1% formic acid (FA) and either 3% acetonitrile (ACN) (solvent A) or 80% ACN (solvent B). The loading solvent was 0.1% TFA and 3% ACN run in Data independent acquisition as previously described (35). The PTM identification and relative abundance was performed in Skyline and Epiprofile 2.0 also as previously described (35).

### Western Blot Analysis

Whole cell lysates were obtained using Laemmli buffer. Samples were separated by SDS-PAGE (12%) and blotted onto poly-vinylidene difluoride (PVDF) membranes (Bio-Rad Laboratories). Blots were incubated overnight at 4°C with antibodies against Actin (Rabbit polyclonal, Sigma-Aldrich) or PTGS2 (Rabbit polyclonal, Cell Signalling). Protein detection was carried out with horseradish peroxidase (HRP)-conjugated secondary antibodies, goat anti-rabbit IgG (Dako, P0448, 1:2500) and ECL substrate (GE Healthcare). Bands were quantified using Image J 1.32 software (v1.8, NIH). The intensity ratio of PTGS2 and actin, were calculated and normalised to the Mock infected samples.

### Metabolic Measurements

The metabolic measurements were performed as previously described (39). Briefly, 14 day old MDM were re-seeded in XF24 cell plates (Agilent Technologies) at 150,000 MDMs/well. Cultures were then challenged with bacteria or mock-infected were washed twice with XF medium supplemented with 4.5 g/L D-glucose, 2.0 mM L-glutamine, 1.0 mM sodium-pyruvate, 100 U/L penicillin and 100 μg/mL streptomycin at pH 7.4 (adjusted with 1.0 M NaOH). Next 630 μL modified XF medium was added to each well and incubated for 1 h at 37·C without CO_2_. 70 μL oligomycin A (15 μM), 77 μL FCCP (20 μM) and 85 μL rotenone (10 μM) plus antimycin A (10 μM) (Sigma-Aldrich) were added to the cartridge injection ports A, B and C, respectively and incubated for 1 h at 37°C without CO_2_. The plate was loaded into the XF24 analyser (Seahorse, Agilent Technologies). After equilibration, the cartridge containing the oxygen sensor, measuring the oxygen consumption rate (OCR) and the cartridge containing the proton sensor, measuring the extracellular acidification rate (ECAR) kinetics were run before and after injecting oligomycin, FCCP and rotenone plus antimycin A, respectively. Data were normalized by total protein.

### DCFDA Assay

For the measurement of reactive oxygen species (ROS) MDMs were washed with PBS three times and incubated in fresh media with the addition of 20 μM 2′,7′-Dichlorofluorescin diacetate (DCFDA, Sigma) for 30min at 37 °C with 5% CO_2_ then washed three times in PBS before being challenged with opsonised *Streptococcus pneumoniae* at an MOI of 10 for 3 hours. The cells were then washed 3 times in PBS and resuspended in 500 μL prior to analysis. Samples were run on an 4 colour FACSCalibur™ (BD Biosciences) using CellQuest Pro version. 10 000 gated events were counted and Forward (FSC) and Side scatter (SCC) were used to define populations. Data analysis was performed in FlowJo™ software (version 10.1r5).

### Statistical Analysis

Statistical analysis was performed using either R for the microarray and proteomic analysis, or for all other experiments in Prism version 7.0c (Graphpad). Data is presented as standard deviation (SD) or standard error of the mean (SEM). For all experiments a minimum of 3 biological replicates was used. Comparison between two paired groups employed a paired t-test, for comparison of 3 or more conditions a one-way analysis of variance (ANOVA) with Tukey’s post-test was performed. For comparison of multiple observations in more than two groups a two-way ANOVA with Tukey’s multiple comparison post-test was performed.

## Results

### Pneumolysin Shapes the Macrophage Transcriptome in Response to Pneumococci

Transcriptional responses to Gram-positive bacteria occur rapidly and are well developed after three hours of bacterial challenge (15, 18). Therefore, we initially analysed transcriptional changes in monocyte-derived macrophages (MDMs) three hours after bacterial challenge with either wild-type or a pneumolysin deficient (Δ*ply*) strain. We confirmed that intracellular viability of pneumococci was equivalent at this time between wild-type and Δ*ply* strains (**Supplemental Figure S1**), thereby excluding potential confounding by different levels of vita-pathogen-associated molecular pattern (40).

**Figure 1:**
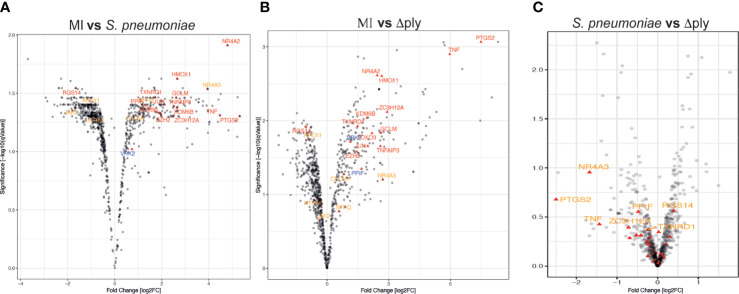
Oxidative stress response pathway terms significantly upregulated. Monocyte derived macrophages (MDMs) were challenged with either *S. pneumoniae*, the isogenic pneumolysin negative mutant (Δ*ply*) or mock infected with phosphate buffered saline (MI) in biological replicates of three. After 3 h incubation the gene expression was measured using Affymetrix arrays. **(A)** volcano plots comparing log2 (fold change) to log10 p value for MI vs *S. pneumoniae* challenged MDMs. **(B)** volcano plots comparing log2 (fold change) to log10 p value for MI vs Δ*ply* challenged MDMs. **(C)** volcano plots comparing log2 (fold change) to log10 p value for *S. pneumoniae* vs Δ*ply* challenged MDMs. The highlighted genes belong to the Oxidative stress response Gene Ontology term and are significantly expressed (q value <0.05). In red are those found in challenge with both strains, in blue the genes that are only found in the response to Δ*ply* challenge and in orange the pneumolysin-dependent genes found after *S. pneumoniae* but not Δ*ply*.

**Figure 2:**
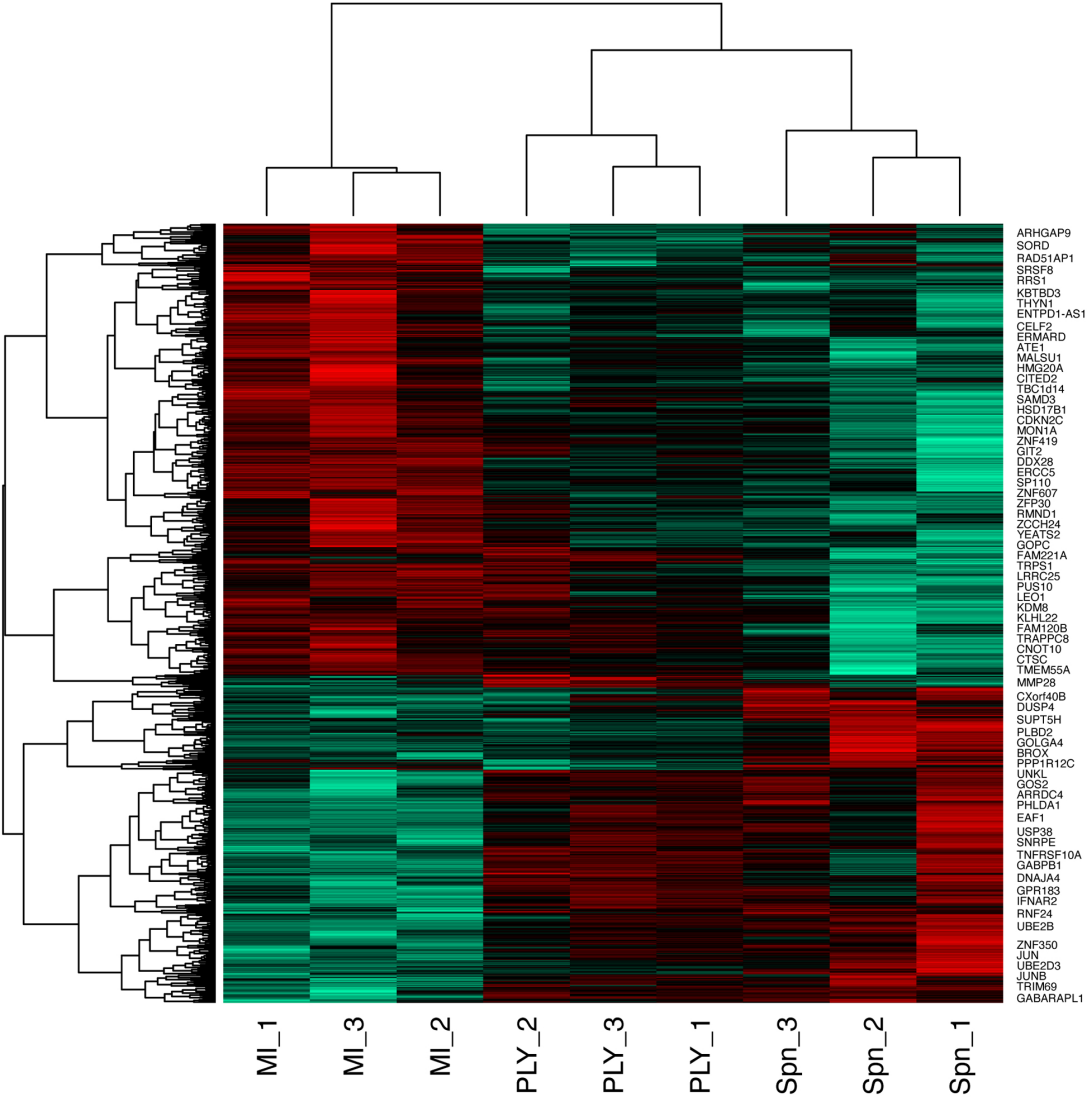
Heatmap of the differentially expressed probes following MDM challenge. Monocyte derived macrophages (MDMs) were challenged with either *S. pneumoniae*, the isogenic pneumolysin negative mutant (Δ*ply*) or mock-infected with phosphate buffered saline (MI) in biological replicates of three. At 3 h the gene expression was measured using Affymetrix arrays. This heatmap represents genes found to be differentially expressed (one-way ANOVA, with F p value <0.05).

We identified 1872 probe-sets that were differentially expressed in response to bacterial challenge with either strain (F value <0.05) and 1553 after multiple test correction false discovery rate (FDR <0.05) (**Supplemental Table S1**). Next, we calculated moderated t tests for each infection, as shown in the Volcano plots in **Figure 1**. The analysis identified 503 probe-sets, which are differentially expressed in a pneumolysin-dependent manner, 506 in an independent manner and 234 only in the absence of pneumolysin (summarised **Supplemental Figure S2** and **Figure 2**). Of the 503 probe-sets differentially expressed in a pneumolysin dependent manner 131 were upregulated and 372 down regulated. The 10 upregulated probes with the highest fold changes included fatty acid binding protein 4 (*FABP4*) involved in cytokine production, and two members of the TNF receptor superfamily (*TNFRSF 9* and *4*) involved in the host immune response, and interleukin 1 receptor antagonist (*IL1RN*) involved in signal transduction (**Supplemental Table S2**). The 234 genes only differentially expressed in the absence of pneumolysin include 96 downregulated probe-sets and 138 upregulated ones. Of the downregulated probes with the 10 highest fold changes four related to DNA binding, LYL1, basic helix-loop-helix family member (*LYL1*), MIS18 binding protein 1 (*MIS18BP1*), ZFP36 ring finger protein like 2 (*ZFP36L2*) and nuclear factor of activated T cells 1 (*NFATC1*). Of the upregulated probes with the highest fold change there were several probes involved in inflammatory responses, cytokine and chemokine signalling such as C-X-C motif chemokine ligand (*CXCL*) -1, and 3, as well as interleukin 1 beta and alpha (**Supplemental Table S2**). Finally, 506 probe-sets were differentially expressed in response to either strain. The probes coding for TNF, prostaglandin- endoperoxide synthase 2 (*PTGS2*) and nuclear receptor subfamily 4 group A member 3 (*NR4A3*) as well as the CXCL8 probe were amongst the 10 upregulated probes with the highest fold change. The downregulated probes included several members of the GTPase IMAP family (*GIMAP1* and *6*).

To provide further insight into the transcriptional responses in MDMs following bacterial challenge we undertook pathway analysis of the differentially expressed genes.

### Pathway Analysis of Differentially Expressed Genes

**Figure 3:**
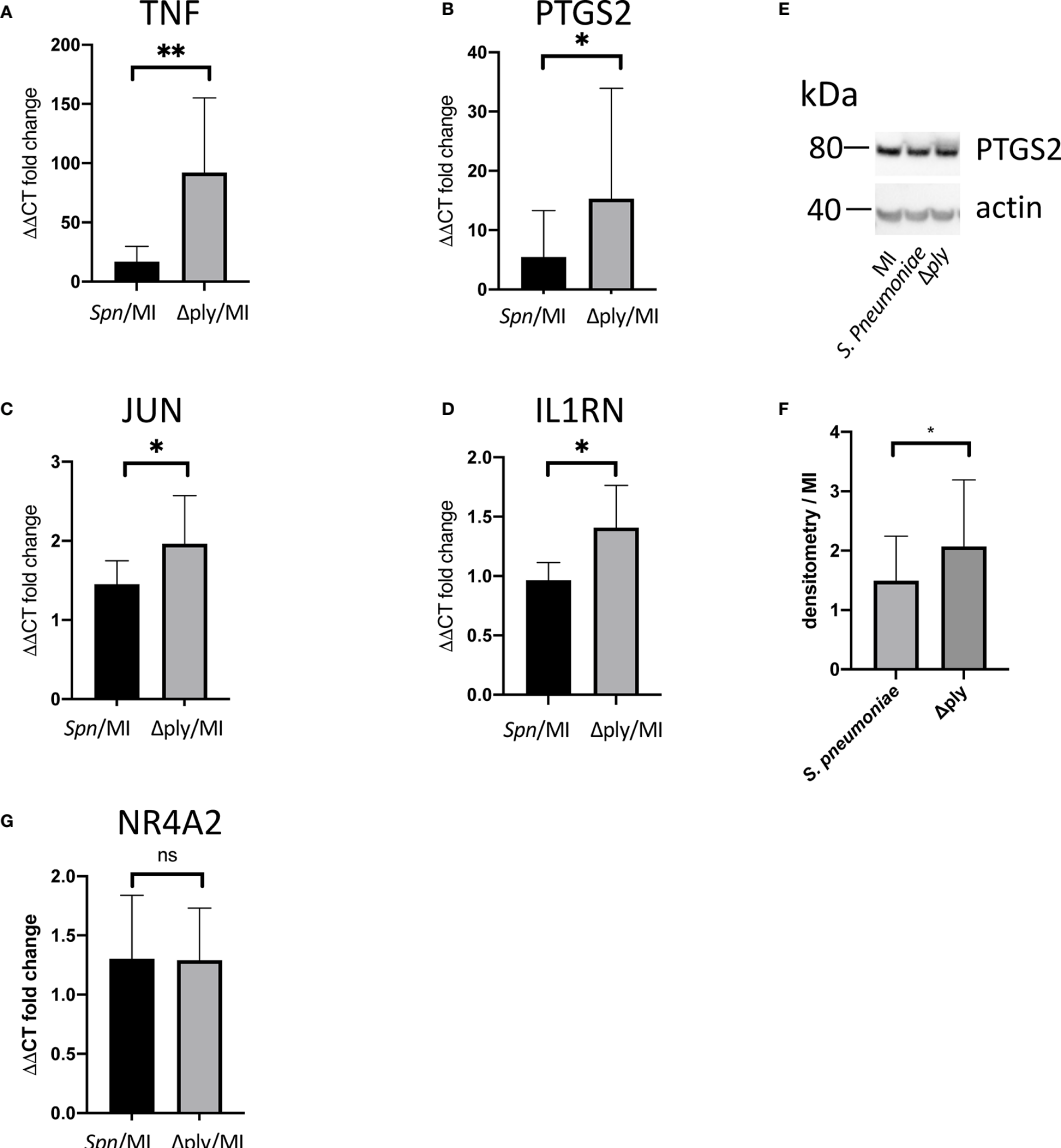
Pneumolysin leads to repression TNF and PTGS2 production. Monocyte derived macrophages (MDMs) were challenged with either *S. pneumoniae*, the isogenic pneumolysin negative mutant (Δ*ply*), or mock-infected with phosphate buffered saline (MI). After 3 h incubation RNA was extracted from MDMs and RT-qPCR performed to measure the abundance of **(A)** TNF, **(B)** PTGS2, **(C)** C-JUN, **(D)** IL1RN and **(G)** NR4A2 mRNA. The Bar chart represents the ΔΔCT fold change for each bacterial challenge demonstrating significantly higher abundance of *TNF, PTGS2, C-JUN* and *IL1RN* mRNA following challenge with Δ*ply*. **(G)** There was no significant difference in the abundance of *NR4A2* mRNA in response to either bacterial challenge. **(E)** A representative western blot after probing with anti-PTGS2 antibody is shown, with actin used as a loading control. The blot is representative of four independent experiments. **(F)** Densitometry was performed to estimate the PTGS2/actin and normalised to mock infected sample. The bar charts represent mean and Standard Deviation, n= 10 for panel **(A, B)**. n=6 for panel **(C)** and n=7 for panel **(D, G)**; *p < 0.05, **p < 0.01.

Gene Ontology (GO) pathway analysis demonstrated that the top ten enriched terms in the MDMs challenged with *S. pneumoniae* were predominantly related to cell metabolism (**Supplemental Figure S3** and **Supplemental Table S3**). As we have previously identified changes in the oxidative stress response in alveolar macrophages in response to challenge with *Streptococcus pneumoniae* (29) we were also interested in the fact that there were a number of terms relating to cellular responses to “stress” [17 in the *S. pneumoniae* challenge and 15 in the Δ*ply* mutant (**Supplemental Table S3**)] and in particular to oxidative stress responses. The differentially expressed genes belonging to the GO term for oxidative stress response were highlighted for each strain (**Figure 1** and **Supplemental Table S4**). These results highlighted that the differentially expressed genes were predominantly upregulated. Moreover, the TNF, heme oxygenase 1 (*HMOX1*) and prostaglandin-endoperoxide synthase 2 (*PTGS2*) genes were strongly upregulated. In order to validate the results of the microarray experiment we measured the levels of mRNA for *TNF, C-JUN, IL1RN*, *PTGS2* and *NR4A2* by qPCR in separate biological replicate (**Figure 3**). This demonstrated that pneumolysin not only blunted the *TNF* response but also *PTGS2* expression. Furthermore, we show that following challenge with *S. pneumoniae* MDM generate reactive oxygen species and leads to alterations in oxidative metabolism (**Supplemental Figure S4**).

In addition to performing Gene ontology analysis we also analysed the differentially expressed genes using eXploring Genomic Relations (XGR), which performs hypergeometric enrichment analysis with background correction using the expression of monocyte derived macrophage cells to give a more cell type specific analysis. Canonical pathway analysis was performed for genes whose expression was upregulated by greater than 2-fold in response to challenge with *S. pneumoniae* (adjusted p value <0.05). This revealed 36 over-represented pathways (**Supplemental Table S5**). Importantly this demonstrated that both the TNF and the NFκB signalling pathways were enriched. Analysis of the 2-fold downregulated terms did not reveal any enriched canonical pathways.

The analysis of the differentially expressed genes whose expression was up-regulated by greater than 2-fold in response to challenge with the Δ*ply* mutant also revealed that the pathways for TNF, NFκB and CD40 signalling were significantly over-represented (**Supplemental Table S6**). This highlights the importance of these pathways in the response to bacterial challenge. It also suggests that the increase in pro-inflammatory signals being released in particular TNF is independent of ply. Of note, we observed a 64-fold increase in the *TNF* mRNA in response to the Δ*ply* mutant strain but only 16-fold increase in response to the parent strain (**Figure 1**).

Additionally, pathway analysis was performed for genes identified by ANOVA as differentially expressed in between the both bacterial challenges (p value <0.05) (**Supplemental Table S7**). This revealed the overrepresentation of a number of signalling pathways such as the ERK1/ERK2 MAPK pathway, integrin-linked kinase signalling, signalling events mediated by PTP1B, SHP2 signalling and Fas signalling pathway. in addition the ATF-2 transcription factor network was overrepresented. Next we performed the GO term analysis on these differentially expressed genes and among the overrepresented terms, we identified histone acetylation, chromatin organisation, and cellular response to oxidative stress (**Supplemental Table S7**).

### Label-Free Quantitative Proteomic Analysis

Having established that there was a transcriptional difference in the host cell response following challenge with *S. pneumoniae* or Δply, further studies were performed to examine the effects of *S. pneumoniae* on the proteome of MDMs.

Label free quantitative mass spectrometry was used to identify differentially expressed proteins following challenge with *S. pneumoniae* or Δply. We identified 1807 proteins in samples challenged with *S. pneumoniae* and 1862 in those challenged with Δply. On average we identified 1812 proteins in each of the 12 samples from 4 biological replicates that we analysed. Of these, we identified 30 that were differentially expressed between the three conditions with an F statistic value of less than 0.05 (summarised in **Table 1**). The results show that 16 proteins are differentially expressed in response to infections with *S pneumoniae*, and 22 in response to Δply. Of the differentially expressed proteins common to both bacterial challenges were Prostaglandin G/H synthase 1 (PTGS1), which is involved with PTGS2 in the production of inflammatory prostaglandins, and Galectin 3-binding protein, which is involved in initiating signalling cascades. In addition the proteins Phosphoglucomutase-1 glucose which participates in breakdown of glucose and Complex III assembly factor LYRM7 which is a mitochondrial chaperone were both differentially expressed highlighting the importance of metabolism in response to infection as illustrated by the microarray pathway analysis results. In addition, although the concordance between transcripts and proteins is generally poor, we observed that both the transcript and the protein abundance for the vacuolar protein sorting 13 C protein (VPS13C), which is associated with mitochondrial function, were differentially expressed.

**Table 1:** Differentially expressed proteins at 6 h following challenge with *S pneumoniae* or Δ*ply*.

		MI vs *S pneumoniae*		MI vs Δ*ply*	
protein name	Gene name	logFC	adj.P.Val	logFC	adj.P.Val
**Prostaglandin G/H synthase 1**	**PTGS1**	**-2.83**	**6.42E-03**	**-1.67**	**2.60E-02**
Serine/threonine-protein kinase OSR1	OXSR1	-2.36	**3.82E-04**	-1.03	**5.59E-02**
Probable global transcription activator SNF2L2/Transcription activator BRG1	SMARCA2/SMARCA 4	-3.69	**3.85E-04**	-0.60	4.99E-01
**Galectin-3-binding protein**	**LGALS3BP**	**-2.36**	**1.26E-03**	**-1.94**	**2.96E-03**
Ubiquitin domain-containing protein 1/2	UBTD2	2.84	**2.48E-03**	0.68	2.95E-01
**Phosphoglucomutase-1 **	**PGM1**	**-2.19**	**1.42E-02**	**-2.69**	**8.14E-04**
**Vacuolar protein sorting-associated protein 13C**	**VPS13C**	**-1.83**	**6.42E-03**	**-2.06**	**1.04E-03**
**Unconventional myosin-Ixb**	**MYOB9B**	**-1.27**	**4.09E-02**	**-2.05**	**1.13E-02**
Probable ATP-dependent RNA helicase DDX46	DDX46	1.69	**6.42E-03**	0.65	2.15E-01
Probable 28S rRNA (cytosine (4447)-C (5))-methyltransferase	NOP2	1.62	**6.42E-03**	0.06	9.32E-01
Coiled-coil domain-containing protein 22	CCDC22	2.54	**1.23E-02**	0.99	1.92E-01
**Sorting nexin-8**	**SNX8**	**1.63**	**2.47E-02**	**-1.22**	**3.36E-02**
**Complex III assembly factor LYRM7**	**LYRM7**	**1.66**	**1.28E-02**	**1.47**	**9.57E-03**
Motile sperm domain-containing protein 2	MOSPD2	-1.29	**1.57E-02**	-0.04	9.32E-01
**Spectrin beta chain, non-erythrocytic 1**	**SPTBN1**	**2.52**	**6.42E-03**	**2.02**	**4.05E-03**
6-phosphogluconolactonase	PGLS	-1.18	**2.47E-02**	-0.06	9.32E-01
**26S proteasome non-ATPase regulatory subunit 5**	**PSMD5**	**2.76**	**6.14E-05**	**1.90**	**1.66E-03**
Ubiquitin conjugation factor E4 A	UBE4A	-1.69	8.34E-02	3.92	7.72E-04
N(G),N(G)-dimethylarginine dimethylaminohydrolase 2	DDAH2	-1.48	5.88E-02	-2.13	1.09E-03
Threonine–tRNA ligase, cytoplasmic	TARS	-1.42	6.93E-02	-2.12	9.57E-03
Haloacid dehalogenase-like hydrolase domain-containing 5	HDHD5	-0.87	2.62E-01	-2.79	1.09E-03
Protein canopy homolog 2	CNPY2	-0.88	8.56E-02	0.67	1.92E-01
Mitochondrial carrier homolog 1	MTCH1	-0.87	1.08E-01	-2.88	3.75E-04
eIF-2-alpha kinase activator GCN1	GCN1	-0.74	1.91E-01	-1.79	1.66E-03
Deoxyribonuclease-2-alpha	DNASE2	-0.65	1.18E-01	0.09	9.00E-01
2’-deoxynucleoside 5’-phosphate N-hydrolase 1	DNPH1	-0.25	7.52E-01	-2.74	1.09E-03
Piezo-type mechanosensitive ion channel component 1	PIEZO1	0.23	7.61E-01	-2.36	1.04E-03
Nuclear pore complex protein Nup155	NUP155	0.39	6.29E-01	2.01	3.27E-03
40S ribosomal protein S15a	RPS15A	0.46	2.68E-01	1.09	1.20E-02
Methylmalonic aciduria type A protein, mitochondrial	MMAA	0.71	2.13E-01	1.94	1.66E-03

Proteins in bold and black are differentially expressed in response to challenge with both strains of bacteria, in red are highlighted the proteins only differentially expressed in response to challenge with Δply and in blue are those that are only differentially expressed in response to challenge with Streptococcus pneumoniae.

We were able to measure the relative abundance of PTGS2 by Western blotting to confirm that these prostaglandin pathways were upregulated by challenge with *S. pneumoniae* and Δ*ply* (**Figure 3**). Furthermore, there was significantly more PTGS2 in response to challenge with Δ*ply*, suggesting that pneumolysin was responsible for blunting of the inflammatory response as was observed with the transcriptomic analysis (**Figure 3**).

Eight proteins were differentially expressed in a pneumolysin-dependent manner, these included serine/threonine protein kinase, oxidative stress response 1 protein (OXSR1) and ubiquitin domain-containing protein 1/2 (UBTD2), and 9 were common to both bacterial strains, including Galectin 3 binding protein which is involved in the host immune responses (41) and the 26S proteasome non-ATPase regulatory subunit 5 (PSMD5). Several of the differentially expressed proteins identified such as PSMD5, UBTD2 and Ubiquitin conjugation factor E4 A (UBE4A) are involved in protein turnover involving a number of pathways known to be important in the regulation of host immune responses (42).

### Pneumolysin Is Responsible for Changes in the Relative Abundance of Histone Post-Translational Modifications

**Figure 4:**
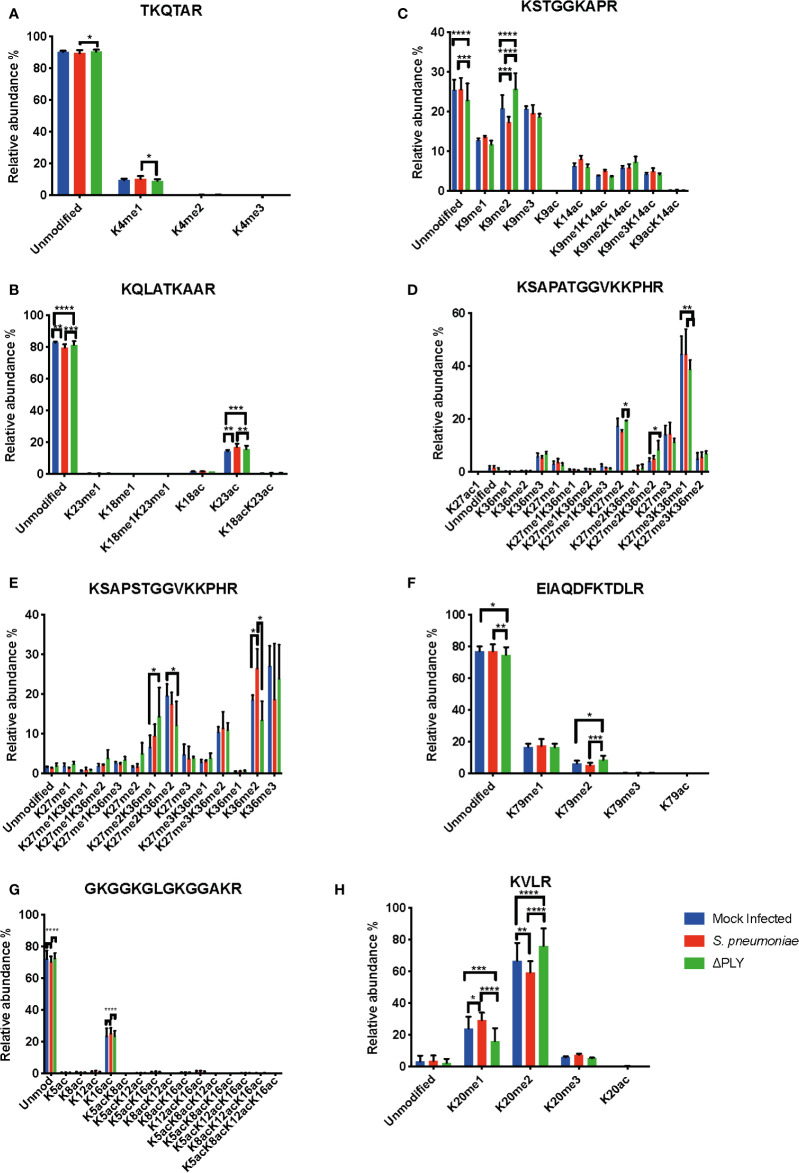
Pneumolysin is responsible for changes in relative abundance of PTMs on histone H3 and H4. Monocyte derived macrophages (MDMs) were challenged with either *S. pneumoniae*, the isogenic pneumolysin negative mutant (Δ*ply*) or mock infected with phosphate buffered saline (MI) in biological replicates of three. After 3 h incubation the histones were extracted and analysed by mass spectrometry and the relative abundance of each post translational modification (PTM) was measured for each peptide. The bar plots represent the relative abundance of each PTM quantified, blue represents the abundance in MI MDMs, green following challenge with *S. pneumoniae* and in red to Δ*ply*. **(A)** Peptide TKQTAR shows an increase in the level of H3K4me1. **(B)** Peptide KSTGGKAPR shows a decrease in the level of H3K9me2 in a pneumolysin-dependent manner. **(C)** Peptide KQLATKAAR shows a relative increase in the level of H3K23ac in response to both bacterial challenges. **(D)** Peptide KSAPATGGVKKPHR shows a decrease in H3K27me2 following challenge with *S. pneumoniae* compared to MI, an increase in the level of H3K27me2K36me2 and a reciprocal drop in H3K27me3K36me1 in response to Δ*ply* challenge compared to MI. **(E)** Peptide EIAQDFKTDLR shows an increase in the relative abundance of H3K79me2 following challenge with Δ*ply*. **(F)** Peptide KSAPSTGGVKKPHR of H3.3 shows an increase in H3.3K36me2 and in H3.3K27me2K36me1 with a reciprocal drop in H3.3K27me2K36me2. (One-way ANOVA, *p < 0.05, **p < 0.01, ***p < 0.001, error bars represent mean and standard deviation). **(G)** Peptide GKGGKGLGKGGAKR shows a relative increase in the level of H4K16ac following challenge with *S. pneumoniae* in comparison to the Δ*ply*. **(H)** Peptide KVLR shows a decrease in the level of H4K20me2 in a pneumolysin-dependent manner compared and a reciprocal increase in the H4K20me1. Conversely for the MDM challenged with Δ*ply* there was a rise in H4K20me2 and a fall in the level of H4K20me1. (n=3. One way ANOVA, *p < 0.05, **p < 0.01, ***p < 0.001, ****p < 0.0001).

Having defined a pneumolysin-dependent alteration of the macrophage transcriptome and proteome in response to *S. pneumoniae*, we sought next to establish if ply also influenced epigenetic changes in response to *S. pneumoniae*. Quantitative mass spectrometry was used to identify changes in the global abundance of histone PTMs following challenge with *S. pneumoniae* or Δ*ply*. Focussing on the most abundant PTMs, methylation and acetylation, on histones H3 and H4, in MDMs in response to bacterial challenge we identified 94 different peptide proteoforms and 18 were found to change in response to bacterial challenge (**Figure 4**). A subset of these changes were ply-dependent, since they were significantly altered in *S. pneumoniae* in comparison to the Δ*ply* mutant (**Supplemental Table S8**). These included significant increases in the relative abundance of H3K4me1 and H3.3K36me2 following challenge with *S. pneumoniae* in comparison to Δ*ply*. In addition, we observed decreases in the relative abundance of H3K9me2, H3K27me2 and H3K79me2 in a pneumolysin-dependent manner compared to both mock infected (MI; blue) and Δ*ply* (**Figure 4**). Moreover, there was a pneumococcal associated ply-independent increase in the level of H3K27me2K36me2 and a reciprocal drop in H3K27me3K36me1. We noted an increase in the relative abundance of H3.3K36me2 in a ply dependent manner and an increase in the relative abundance of H3K23ac and an increase in H3.3K27me2K36me1 with a reciprocal drop in H3.3K27me2K36me2 in response to both bacterial challenges.

Further analysis of histone H4 showed a significant increase in the abundance of acetylation on K16 (H4K16ac) in response to infection with either strain (**Figure 4**). In addition, there was also a decrease in the dimethylated form of K20 (H4K20me2) and a reciprocal increase in the monomethylated form (H4K20me1) in a pneumolysin-dependent manner.

### Pneumolysin Blunts Inflammatory Signalling During Early Infection

**Figure 5:**
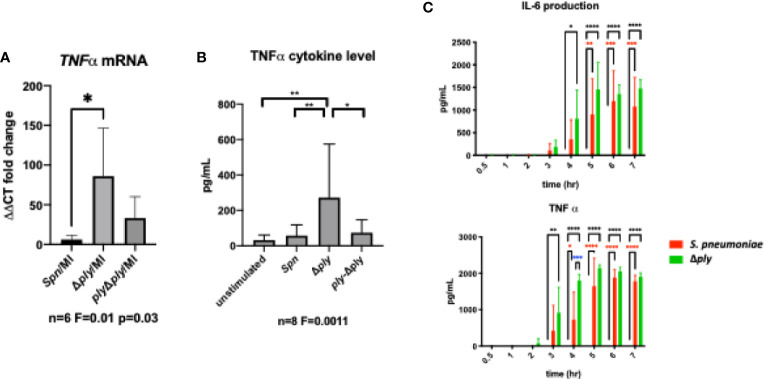
Pneumolysin leads to blunted TNFα release by repressing mRNA production. Monocyte derived macrophages (MDMs) were challenged with either *S. pneumoniae*, the isogenic pneumolysin negative mutant (Δ*ply*), the isogenic pneumolysin negative mutant with reconstituted pneumolysin (*ply*-Δ*ply*). or mock-infected with phosphate buffered saline (MI). **(A)** After 3 h incubation RNA was extracted from MDMs and RT-qPCR performed to measure the abundance of TNFα mRNA. The Bar chart represents the ΔCT fold change for each bacterial challenge demonstrating significantly higher abundance of TNFα mRNA following challenge with Δ*ply*. (n= 6 One-way ANOVA F statistic =0.01 and p value <0.05. **(B)** At 3 h TNFα concentration were measured in the supernatants of MDMs challenged with each strain. The Bar chart demonstrate the significantly raised level of TNFα in response to challenge with △*ply* (n=8, one way ANOVA F statistic = 0.0011, p<0.05). **(C)** TNFα and IL-6 cytokines were measured in supernatants following challenge with *S. pneumoniae* or △*ply* at 0.5, 1, 2, 3, 4, 5, 6, 7 h. The bar charts represent mean and Standard Deviation of 3 biological replicates run in technical duplicates are shown demonstrating a significant difference in the rise of TNF-α between the two strains at 4 h, (black line highlights differences between control and Δ*ply*, red line between control and *S pneumoniae*, blue line *S pneumoniae*, and Δ*ply*, *p < 0.05, **p < 0.01, ****p < 0.0001).

In order to establish the consequences of these changes to the transcriptome and epigenome, we next selected one prominent immune signature regulated by pneumolysin, TNF-α expression, and established this as a key component of the early immune response to *S. pneumoniae* (43). Using qRT-PCR we measured the abundance of TNFα mRNA in infected MDMs. There was a significant increase in the TNFα mRNA level following infection with the ply deficient mutant but not the parent strain or the reconstituted mutant (**Figure 5A**). Next, we measured the amount of secreted of TNFα in MDM supernatants following 3 h of bacterial challenge (**Figure 5B**). We found that TNFα levels were significantly higher following challenge with the Δ*ply* mutant suggesting that ply initially blunts TNF-α expression.

Finally, we measured the concentration of TNFα and IL-6 over time using ELISA assays to determine if this initial anti-inflammatory effect was maintained (**Figure 5C**). The results showed that the difference seen in the release of TNF-α, was only observed at an early time window, 3-5 h after exposure to bacteria. In addition there was a similar trend observed with the inflammatory cytokine IL-6. This suggests that the anti-inflammatory effect of pneumolysin only plays a role over a defined time period.

### Histone Deacetylase Inhibitors Reduce TNFα Cytokines in Response to Infection

**Figure 6:**
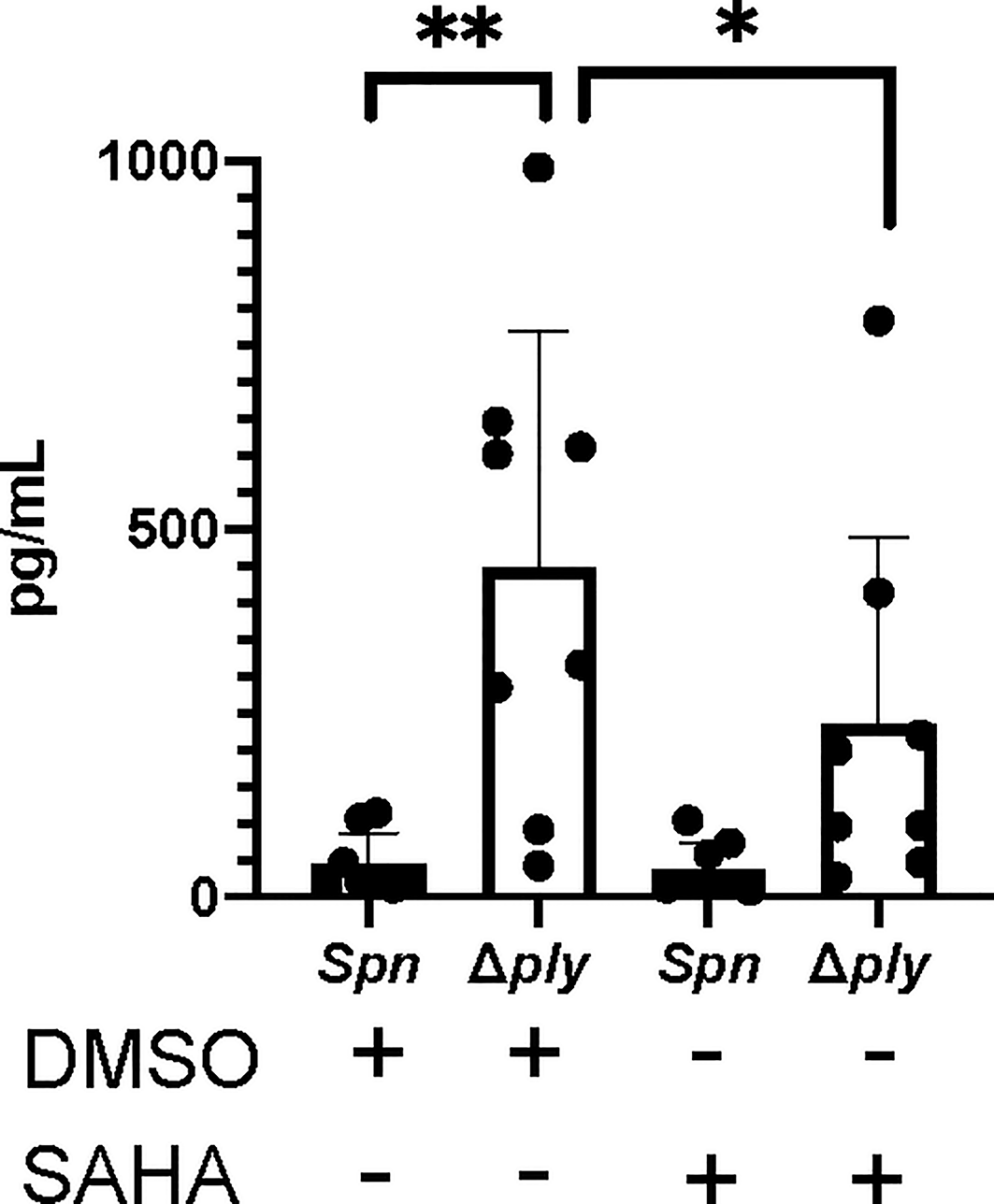
The HDAC inhibitor Vorinostat suppresses TNFα levels to similar levels as pneumolysin. Monocyte derived macrophages (MDMs) were challenged with either *S. pneumoniae*, the isogenic pneumolysin negative mutant (Δ*ply*) or mock infected with phosphate buffered saline (MI) in the presence of 3 μM vorinostat (SAHA) or vehicle control (0.5% DMSO). The Bar chart and standard deviation represents the levels of TNFα in the supernatants demonstrating that suppression of TNFα can be mimicked by SAHA to similar levels as in the presence of ply. (n=8 **p < 0.007 *p < 0.05).

Having previously demonstrated that pneumolysin both modifies macrophage transcriptome and epigenome we next tested if epigenetic modifications could alter key ply-associated immune responses. As a proof of concept, we pre-treated MDMs with the histone deacetylase inhibitor (HDACi) vorinostat (SAHA) or vehicle control (DMSO) following challenge with *Streptococcus pneumoniae* or Δ*ply* mutant and quantified TNFα release (**Figure 6**). As previously shown, challenge with the Δ*ply* mutant strain resulted in significantly increased TNFα levels as compared to the parental strain, but this level was significantly decreased in the Δ*ply* mutant challenged cells by pre-treatment with vorinostat. This suggests that the epigenetic modifications we observed as being induced by ply exposure could have functional consequences to immune responses, as illustrated for TNF.

## Discussion

Using a range of systems level approaches we have demonstrated that pneumolysin exerts a strong influence over the host response to *S. pneumoniae* at the transcriptomic, proteomic and epigenetic level. We have shown that 503 probes were differentially expressed in pneumolysin-dependent manner in association with global modifications in histone PTMs. These changes are associated with widespread changes in macrophage metabolism, oxidative stress responses and cytokine signalling and result in differential expression of key immune proteins, including TNF-α and IL-6. Crucially we show that use of a HDAC inhibitor to modify the epigenome is sufficient to reprise the reduction in TNF identified as occurring in response to pneumolysin.

Our results on the transcriptional response of macrophages to pneumolysin are consistent with previous transcriptomic analysis in undifferentiated THP-1 cells that showed 142 genes to be differentially expressed in a ply dependent manner and 40 to be ply independent (15). Our study in primary cells, identifies many more genes differentially regulated enabling more in-depth analysis of changes at a pathway level. A number of differentially expressed genes are involved in the immune response such as *TNF* and *HMOX1*. Pathway analysis of the differentially expressed probes highlighted the importance of metabolic pathways in response to infection, and in particular the role for oxidative stress responses. The host’s oxidative stress responses have been highlighted as playing a key role in the host response to *S pneumoniae* in lung epithelial cells (44). Importantly, this module is upregulated in alveolar macrophages (AM) and plays a key role in preserving responses such as phagocytosis which may otherwise be altered by dysregulated oxidative stress in AM and MDM (7, 45). Furthermore, nuclear factor erythroid 2 (NRF2) plays a pivotal role in the protection against lung injury (46), reduces lung inflammation after intratracheal instillation of LPS and decreases mortality in systemic models of inflammation (47). Crucially, NRF2-regulated pathways are amenable to therapeutic manipulation as evidenced by improved AM phagocytosis in patients with chronic obstructive pulmonary disease following *ex vivo* treatment with NRF2 agonists and also in a mouse model of cigarette smoke associated impairment of bacterial clearance in the lung (48). The XGR pathway analysis following bacterial challenge further highlighted NFkB, NOD-like receptor and TNF signalling as enriched pathways emphasising their importance in the host’s response to bacteria.

The label-free quantitative proteomic analysis revealed a small number of significantly differentially expressed proteins, but substantiated involvement of several of the pathways identified at the transcriptomic pathway level. Examples of proteins linked to these pathways included an increase in the coiled-coil domain containing protein 22 (CCDC22), that plays an essential role in NFκB signalling and whose depletion leads to blockade of signalling (48, 49). We also noted downregulation of the oxidative stress response protein 1 (OXSR1) involved in the oxidative stress response (50) in a ply-dependent manner.

The quantitative proteomic analysis identified a number of pneumolysin-dependent differentially expressed proteins involved in the regulation of gene transcription, protein translation, and modulation of signalling pathways such as the NFkB pathway, which are predicted to regulate innate immune responses to bacteria.

Finally, the quantitative mass spectrometry analysis of histone PTMs in response to *S pneumoniae* was used to identify and quantify 94 different peptide proteoforms. Of these, there were 5 whose relative abundance changed in a pneumolysin dependent manner. This is to our knowledge the first time that this approach has been applied to study the changes in relative abundance of histone PTMs in primary MDMs in response to *S. pneumoniae.* The advantages of mass spectrometry analysis used in this study were emphasised by the identification of combinatorial marks on both H3 and H3.3 that would not have been possible using antibody-based approaches. Interestingly several of these modifications are associated with activating or repressing gene transcription. Although some histone PTMs are well characterised in a number of systems, their exact function in the context of infections is yet to be fully described. It is likely to vary between cell types. Furthermore, individual PTMs will not act in isolation but rather be part of a combinatorial code fine tuning responses to environmental stressors, such as to bacterial infection and at later time points after exposure to consequences such as DNA damage. Nevertheless, in light of the changes observed in response to challenge with *S. pneumoniae* (*e.g.* increases in H3K4me1, H3K23ac or H4K16ac and decreases in H3K9me2 or H3K79me2) it is possible that these changes represent the removal of repressive marks and the increase in marks associated with active gene transcription allowing fine tuning of the innate immune host response to bacteria to occur. This highlights the important role played by PTMs in the response to infection and therefore offer the potential for the novel use of therapeutic approaches involving immunomodulation of host responses. However, our current study does not establish a direct connection between the histone PTMs and the functional consequences identified. Future work to identify these such as Chromatin Immunoprecipitation and sequencing will enable the integration of histone PTMs changes at the gene level and in combination with further work to explore the epigenetic regulation of the oxidative response and cellular metabolism in response to infection may allow the development of more targeted approach to immunomodulation.

The functional consequences of the net changes to transcriptional responses involving inflammatory cell signalling and regulation of protein expression were examined focusing on TNF-α as an exemplar early response cytokine that plays a key role against *S. pneumoniae* (43). We selected this cytokine since ply induced a significant temporal reduction in production of TNF-α, in conjunction with decreased production of IL-6. Our demonstration that pneumolysin inhibits the early production of TNF-α is consistent with the findings others have reported in dendritic cells and murine AM (16). We were able to demonstrate that changes in the epigenetic landscape are sufficient to reverse the maximal induction of TNF-α production observed following challenge with the ply-deficient mutant. These experiments, using a HDACi, suggest that blunting of TNF-α release by MDM following challenge with a pneumococcal strain expressing ply could be mediated through epigenetic mechanisms. This is important since early response cytokine generation is critical to the outcome of infection in *S. pneumoniae* and any modulation through reshaping the epigenetic landscape would be anticipated to have major consequences to the outcome of infection. Of note, the HDAC inhibitor Cambinol has been shown to inhibit TNF and IL-6 secretion in bone marrow-derived macrophages stimulated with LPS and was associated with greater survival in a murine lethal endotoxemia model and in response to *Klebsiella pneumoniae* challenge (51). In this case the therapeutic intervention was thought to limit excessive cytokine responses whereas in the case of ply we would suggest it may limit early cytokine responses required to enhance early pathogen control. This emphasizes that in line with other aspects of host modulation through epigenetic manipulation would require careful calibration.

In summary our findings using a range of systems levels approaches show that pneumolysin modifies the early host response of macrophages to *S. pneumoniae* through modification of inflammatory responses, resistance to oxidative stress and metabolic responses. We observe that these transcriptional responses and the differential protein expression are associated with global changes in histone PTMs. We show alteration of the epigenetic landscape has functional consequences to key immune responses such as TNF-α production and suggest that ply exerts these effects through epigenetic modulation and regulation of histone acetylation status. Our results also hint at a potential route by which these early host responses can be altered to improve responses to infection through the use of agents that modulate the enzymes that induce the signature pathogen-mediated epigenetic marks that adversely impact host responses.

## Data Availability Statement

The datasets presented in this study can be found in online repositories. The data presented in the study are deposited in the ArrayExpress repository, accession number E-MTAB-9055: https://www.ebi.ac.uk/arrayexpress/experiments/E-MTAB-9055/.

## Ethics Statement

The studies involving human participants were reviewed and approved by South Sheffield Regional Ethics committee (07/Q2305/7). The patients/participants provided their written informed consent to participate in this study.

## Author Contributions

JC: conceptualization, funding acquisition, investigation, writing original draft, and review and editing. AA: investigation and review and editing. RE: formal analysis and review and editing. TM: investigation and review and editing. MD: funding acquisition, conceptualization, supervision, and writing and editing. DD: conceptualization, funding acquisition, supervision, and writing and editing. All authors contributed to the article and approved the submitted version.

## Funding

JC was supported by The Wellcome Trust who funded this work (WT104437/Z/14/Z). DD was supported by MRC cross council AMR funding initiative SHIELD consortium MR/N02995X/1. MD acknowledges support from BBSRC UK (award no. BB/M012166/1). The funders had no role in the study design, data collection, decision to publish, or preparation of the manuscript.

## Conflict of Interest

The authors declare that the research was conducted in the absence of any commercial or financial relationships that could be construed as a potential conflict of interest.

## Supplementary Material

Supplementary Figure 1 Viable intracellular bacteria following 3 h challenge with *S. pneumoniae*, Δ*ply* or *ply*-Δ*ply* mutants. Monocyte derived macrophages (MDMs) were challenged with either *S. pneumoniae*, the isogenic pneumolysin negative mutant (Δ*ply*) or the isogenic pneumolysin negative mutant with reconstituted pneumolysin (*ply*-Δ*ply*). At 3 h the number of viable intracellular bacteria was determined. The results are expressed as mean and log_10_ of cfu/mL and represented as bar chart with standard deviation. (n=3, one way ANOVA p=0.99)

Supplementary Figure 2 Venn Diagram representing the overlap in differentially expressed genes.

Supplementary Figure 3 Gene ontology biological processes enriched terms show stress responses and metabolism are over-represented. Monocyte derived macrophages (MDMs) were challenged with either *S. pneumoniae*, the isogenic pneumolysin negative mutant (Δ*ply*) or mock-infected with phosphate buffered saline (MI) in biological replicates of three. At 3 h the gene expression was measured using Affymetrix arrays. The bubble plot shows the top ten enriched Gene Ontology (GO) biological processes terms in both the pneumolysin mutant and the parental strain analysis. The bubble size and colour correspond to the number of genes that have mapped to the GO term. The bubbles are plotted along the x axis according to the –log10 p value for the enrichment.

Supplementary Figure 4 Metabolic profile of MDMs following S. pneumoniae challenge. MDMs were challenged with *S. pneumoniae* or mock-infected with phosphate buffered saline (MI) in four biological replicates for 3 hours. The extracellular acidification rate (ECAR) and mitochondrial oxygen consumption rate (OCR) was measured by the Seahorse X24 extracellular flux analyser. **(A)** representative plots for the OCR kinetic data **(B)** basal OCR, **(C)** maximum respiration capacity, **(D)** DCFDA Reactive Oxygen Species measured by flow cytometry. Data are shown as mean ± SD, n=4. Paired t test, *p<0.05. **p<0.01 ***p<0.001

Supplementary Table 2 10 highest up-regulated and 10 down-regulated differentially expressed probes following challenge of MDMs with either *S. pneumoniae* or δ*ply*. Differentially expressed probes referenced in the text are highlighted in bold.

Supplementary Table 3 Gene Ontology Biological Processes pathway analysis of up-regulated differentially expressed probes following challenge of MDMs either *S. pneumoniae* or Δ*ply*. Gene Ontology pathways of interest that are referenced in the text are highlighted in bold.

Supplementary Table 4 Summary of differentially expressed genes related to the Oxidative stress GO term. In bold are the terms *TNF, PTGS2* and *HMOX1* referenced in the text.

Supplementary Table 5 Canonical pathway analysis of up-regulated differentially expressed probes following challenge of MDMs with *S. pneumoniae* using XGR. In bold are the TNF and NF-KB pathways referenced in the text.” to the legend for table S5.

Supplementary Table 7 Canonical and GO Biological processes pathway analysis of differentially expressed probes between both bacterial challenges. In bold are the Gene Ontology terms (histone acetylation, cellular response to oxidative stress and chromatin organisation) referenced in the text.
