# Supplementary figures and images for: Pneumolysin Is Responsible for Differential Gene Expression and Modifications in the Epigenetic Landscape of Primary Monocyte Derived Macrophages
Source: Front Immunol. 2021 May 11;12:573266. doi: 10.3389/fimmu.2021.573266 (PMC8145618; doi:10.3389/fimmu.2021.573266)

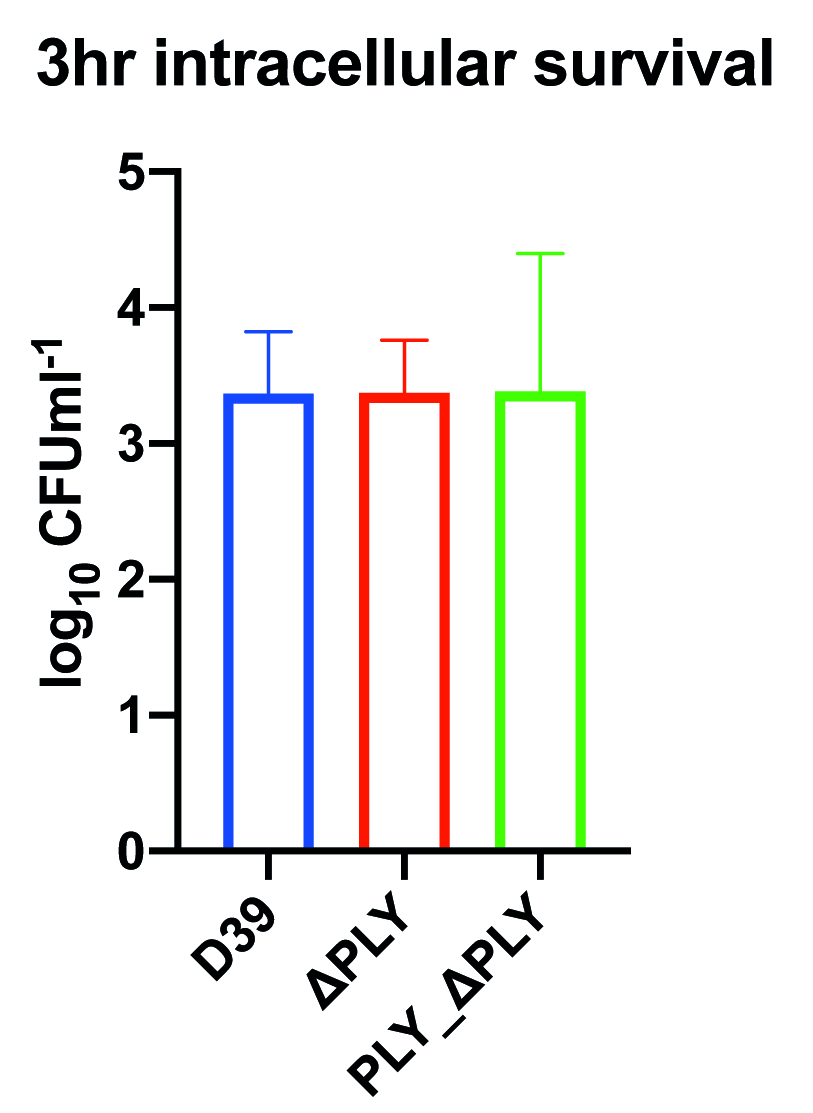

Supplement: Supplementary Figure 1 — Viable intracellular bacteria following 3 h challenge with S. pneumoniae, Δply or ply-Δply mutants. Monocyte derived macrophages (MDMs) were challenged with either S. pneumoniae, the isogenic pneumolysin negative mutant (Δply) or the isogenic pneumolysin negative mutant with reconstituted pneumolysin (ply-Δply). At 3 h the number of viable intracellular bacteria was determined. The results are expressed as mean and log10 of cfu/mL and represented as bar chart with standard deviation. (n=3, one way ANOVA p=0.99) [file Image_1.tif]

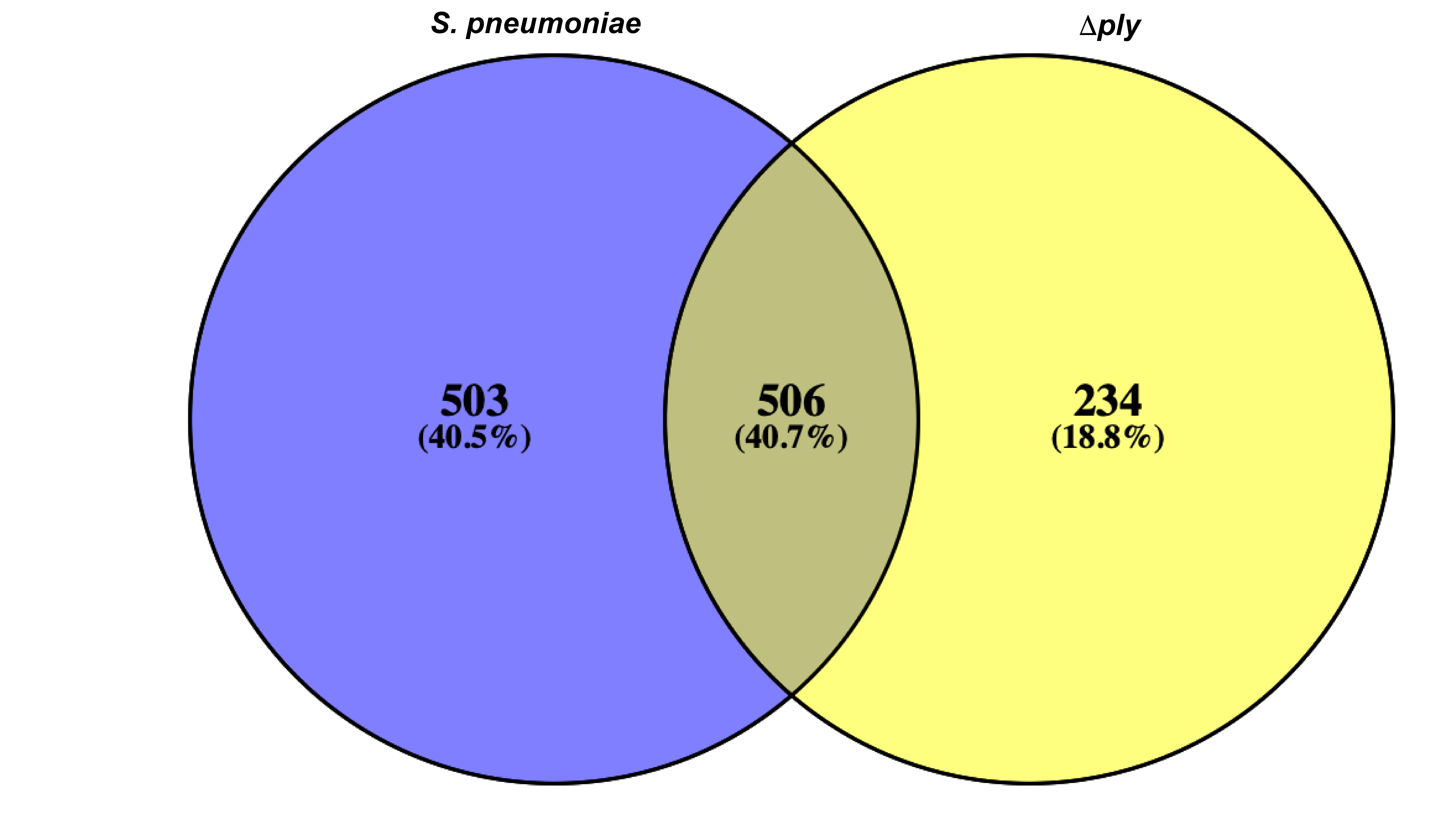

Supplement: Supplementary Figure 2 — Venn Diagram representing the overlap in differentially expressed genes. [file Image_2.png]

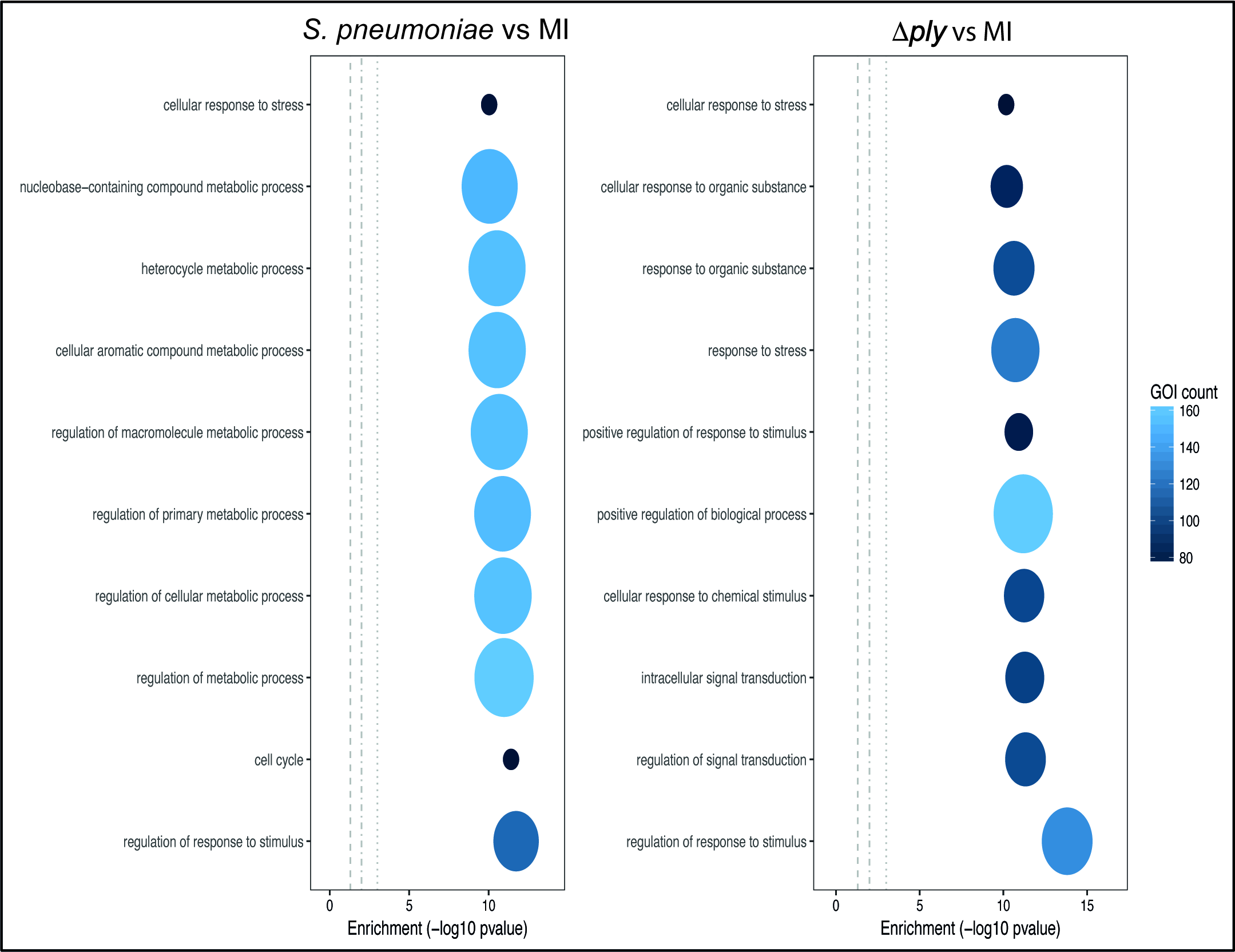

Supplement: Supplementary Figure 3 — Gene ontology biological processes enriched terms show stress responses and metabolism are over-represented. Monocyte derived macrophages (MDMs) were challenged with either S. pneumoniae, the isogenic pneumolysin negative mutant (Δply) or mock-infected with phosphate buffered saline (MI) in biological replicates of three. At 3 h the gene expression was measured using Affymetrix arrays. The bubble plot shows the top ten enriched Gene Ontology (GO) biological processes terms in both the pneumolysin mutant and the parental strain analysis. The bubble size and colour correspond to the number of genes that have mapped to the GO term. The bubbles are plotted along the x axis according to the –log10 p value for the enrichment. [file Image_3.tif]

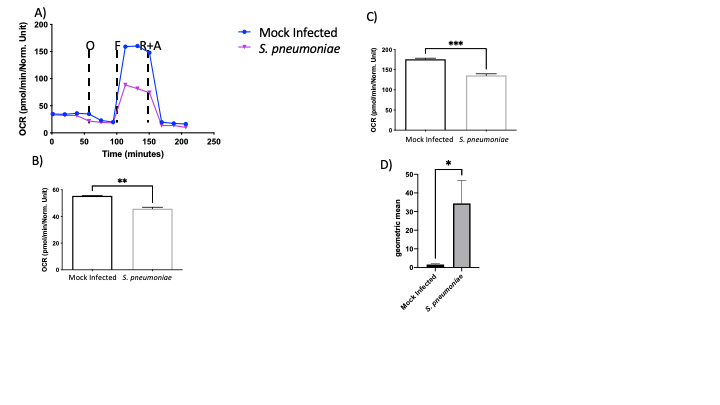

Supplement: Supplementary Figure 4 — Metabolic profile of MDMs following S. pneumoniae challenge. MDMs were challenged with S. pneumoniae or mock-infected with phosphate buffered saline (MI) in four biological replicates for 3 hours. The extracellular acidification rate (ECAR) and mitochondrial oxygen consumption rate (OCR) was measured by the Seahorse X24 extracellular flux analyser. (A) representative plots for the OCR kinetic data (B) basal OCR, (C) maximum respiration capacity, (D) DCFDA Reactive Oxygen Species measured by flow cytometry. Data are shown as mean ± SD, n=4. Paired t test, *p<0.05. **p<0.01 ***p<0.001 [file Image_4.tiff]
